# The Presence and Behaviour of Birds at Two Prescribed Fires in Sweden

**DOI:** 10.1002/ece3.73762

**Published:** 2026-05-30

**Authors:** Ivo Jacobs

**Affiliations:** ^1^ Department of Philosophy and Cognitive Science Lund University Lund Sweden

**Keywords:** behaviour, birds, conservation, fire

## Abstract

Fire has shaped terrestrial ecosystems for hundreds of millions of years. Human activities dramatically shift natural fire regimes, leading to adverse conservation impacts that are projected to increase in severity. Many animals appear ill‐equipped to face these drastic changes, and they may be unable to adapt rapidly enough. In contrast, behavioural plasticity could offer a faster solution to achieve a suitable balance between avoiding the risks and maximising the opportunities of fire. Birds from fire‐prone regions have been observed to exploit fire and burned landscapes for various benefits. However, it is poorly understood how birds from other regions respond to active fire. Here, I observed the presence of 17 bird species at two prescribed fires in Sweden. They appeared to largely ignore it, with some even singing close to fire and smoke. Future studies should systematically examine bird abundance and behaviour across different fire ecologies, which would provide invaluable insights into how the immediate response to active fire may be shaped through adaptations, senses, and learning, eventually leading to effective conservation strategies in an increasingly fiery world.

## Graphical abstract

Shifting fire regimes pose immediate threats to animal conservation. Swedish birds appear to mostly ignore prescribed fire, and some species even continue to sing. Behavioural plasticity towards fire is poorly understood, but may prove to be an essential tool in animal conservation.

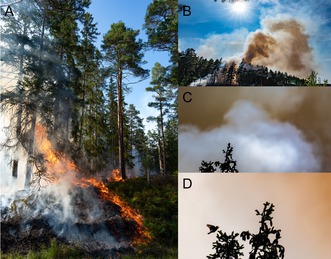

## 1 Introduction

Fire has played a dual role as a creative and destructive force for hundreds of millions of years. The Anthropocene is characterised by shifted fire regimes through activities such as land modification and climate change, which have altered the frequency, intensity, severity, and scale of wildfires (Jones et al. 2023; Kelly et al. 2020; Pyne 2020; van Eeden et al. 2020). Humans kindled this era of extreme fire behaviour or ‘Pyrocene’ (Pyne 2020), yet are not alone in suffering the consequences. Shifting fire regimes already endanger 14% to 19% of threatened terrestrial vertebrates (Kelly et al. 2020), as illustrated by 17 million deaths in the Brazilian Pantanal in 2020 (Tomas et al. 2021) and the habitat of three billion animals ravaged during Australia's Black Summer (van Eeden et al. 2020). Intergovernmental bodies warn that these threats will increase in the future, with an estimated 50% increase in global wildfires by 2100 (IPCC 2022; UNEP 2022).

How do animals cope with such drastic environmental change? Evolutionary adaptation may be too slow to equip animals with an appropriate response when the threat was absent or insignificant in their evolutionary history (Nimmo et al. 2021). In contrast, behavioural plasticity can operate orders of magnitude faster, thereby offering effective mitigation strategies against novel ecological challenges (Candolin et al. 2023; Greggor et al. 2016, 2020; Jones et al. 2023). However, fire‐related mortality is poorly understood (Jolly et al. 2022), let alone how it is affected by behaviour. Until recently, the immediate response of animals to fire has been largely overlooked in fire ecology, despite its capacity to signal conservation concerns early on (Cerini et al. 2023; Michel et al. 2023; Nimmo et al. 2021; van Eeden et al. 2020).

While most animals avoid fire, others associate or interact with it (Jacobs 2021). Recently burned habitats can improve foraging, travelling, thermoregulation, reproduction, camouflage, and predator detection (Jones et al. 2023; Hanzawa et al. 2024; Herzog et al. 2016, 2022; Hovick et al. 2017; Newsome and Spencer 2022; Nimmo et al. 2021). Active fire can provide similar opportunities, particularly for predatory species that hunt fleeing animals (Bonta et al. 2017; Hovick et al. 2017; Komarek 1969). However, fire‐naïve animals may fail to detect fire or respond appropriately, and suffer disproportionate casualty rates (Nimmo et al. 2021). The success of fleeing animals likely hinges on their ability to detect fire, move quickly, and find shelter (Michel et al. 2023; Nimmo et al. 2021; van Eeden et al. 2020). Appropriate responses form an optimal balance between avoiding risks—such as unnecessary energy expenditure and fire‐induced morbidity—and maximising opportunities—such as finding food and mates (Michel et al. 2023; Nimmo et al. 2021).

The immediate response of birds to fire has been documented mainly in fire‐prone regions. South African insectivorous and predatory birds gather at prescribed fires, foraging in front of the advancing fire and on the ground (Dean 1987; Barnard 1987; Komarek 1969; Pons et al. 2003; Bouwman and Hoffman 2007). Bald ibises ( *Geronticus calvus* ) preferentially forage on grassland immediately after a burn, when both living and dead arthropods are more numerous (Manry 1984). Bonelli's eagles ( *Aquila fasciata* ) in Spain either ignore fire or avoid it for a few days (Morollón et al. 2022). In the Brazilian Pantanal, predatory and scavenging species arrive at prescribed fires within an hour after ignition, and savannah hawks ( *Heterospizias meridionalis* ) hunt at active fire (Dainezi and Ribeiro 2025). Examples from North America show that various raptors are attracted to prescribed fires, where they hunt fleeing prey and scavenge on recently burned terrain (Caven et al. 2023; Hingtgen 2000; Hovick et al. 2017; Komarek 1969; Murphy and Smith 2007; Parker 1971; Smallwood et al. 1982; Stewart et al. 2024; Tewes 1984). Granivores also forage on blackened ground, where seeds may be easier to detect (Hingtgen 2000). Western cattle egrets (*Ardea ibis*) even forage within a meter of fire and fly through dense smoke (Komarek 1969; Smallwood et al. 1982). They prefer to forage in recently burned habitat, where their prey capture efficiency is higher—likely because live prey becomes more exposed (Venne and Frederick 2013). Similarly, Australian raptors, generalists, and insectivores forage at fires and their aftermath (Bonta et al. 2017; Braithwaite and Estbergs 1987; Corbett et al. 2003; Komarek 1969; Woinarski 1990, 1999; Woinarski and Recher 1997). Some raptors even carry burning sticks (Bonta et al. 2017) or singe their feathers when flying too close to fire (Braithwaite and Estbergs 1987).

This study reports the presence and immediate response of birds to two prescribed fires in Sweden, where this question received little attention, similar to other regions where wildfires are less common. In an effort to restore a more natural fire regime and increase biodiversity, policymakers and landowners have started intentionally setting small, low‐intensity forest fires every 10–50 years, which are more frequent and less severe than larger wildfires that naturally occurred every 70–120 years (Niklasson 2011; Plathner et al. 2026; Sjöström and Granström 2023). The main goals of these prescribed fires are to increase forest heterogeneity and the amount of deadwood and fire scars on trees—essential resources for saproxylic animals, fungi, and lichens. As such, prescribed fires are intended to improve forest resilience and biodiversity of fire‐dependent species, many of which have declined after centuries of fire suppression (Gustafsson et al. 2019; Länsstyrelsen 2026; Plathner et al. 2026; Ramberg et al. 2026).

Since fires—whether natural or prescribed—are projected to become more common in Sweden (Sjöström and Granström 2023), understanding how wildlife responds to fire over time will have conservation implications. Studies on the long‐term ecological effects of fires in Sweden show that previously burned forests host a more diverse avian community (Edenius 2011; Gustafsson et al. 2019; Versluijs et al. 2017). In terms of immediate responses, observers have anecdotally reported that raptors, black woodpeckers ( *Dryocopus martius* ), and grey‐headed woodpeckers ( *Picus canus* ) forage by prescribed fires (Steen 2021; Emil Persson and Per Gustafsson, pers. comm.).

## 2 Methods

The observations took place at prescribed burns performed by the Life2Taiga project (Länsstyrelsen 2026). The aim of these burns was to create low‐intensity surface fires to produce a mosaic landscape and to increase the biodiversity of fire‐dependent taxa. Fires were lit with drip torches by multiple people in a coordinated fashion. The edges of the prescribed burn area were actively watered to ensure the fire remained restricted to the planned borders. Active monitoring continued for several days afterwards to extinguish any remaining fires. Detailed plans for where, when, and how to burn were made in advance by the regional project leadership, but the final approving decisions were made only the day before if the weather forecast proved favourable (Länsstyrelsen 2026). This presented logistical challenges and prevented me from attending more prescribed burns or performing observations before burns as a control condition.

Both burns were in conifer‐dominated Natura 2000 areas, which are nature protection areas designated by the European Union. I collected data away from the ignition point and just outside and on a corner of the perimeter, where I remained throughout the day. All bird species seen or heard were recorded. Because this was a descriptive investigation to potentially lay the groundwork for a more detailed study, species abundance was not quantified. I used a camera (Nikon Z8) with telephoto lens (Nikkor Z 800mm f/6.3 VR S) to capture images when possible.

**FIGURE 1:**
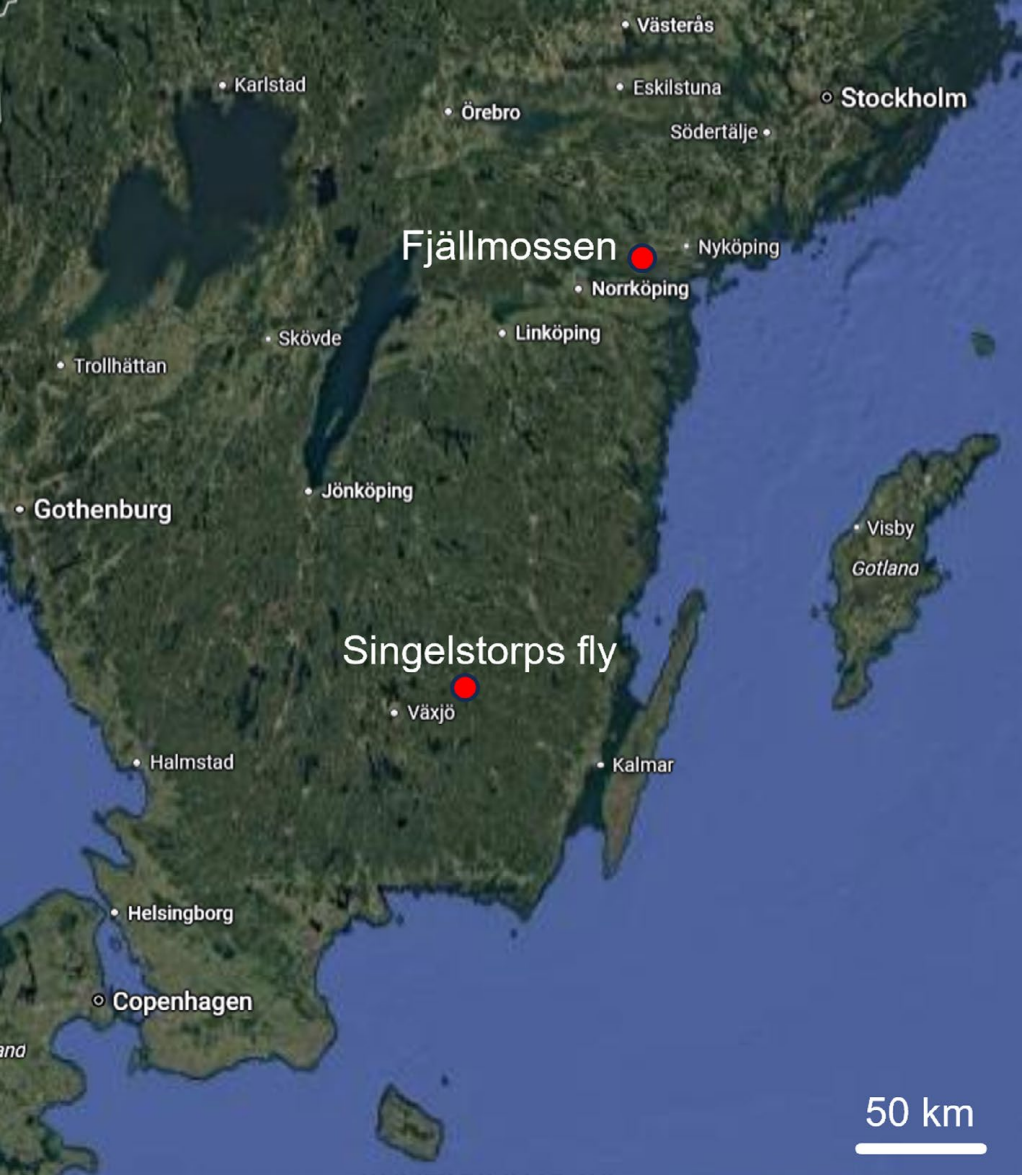
A map of southern Sweden showing the locations of the prescribed burns (adapted from Google Maps).

The fire at Fjällmossen (Figure 1; 58°41′30.0″ N 16°32′33.0″ E) was lit at 13:20 on 30 May 2024. The observations commenced 20 min later until the start of active extinguishment at 19:45, resulting in six hours of observations. In total, 13.5 ha burned as planned. The next day, I walked through the burnt area from 11:00 until 14:00 to record additional observations. There was still much smoke and efforts to extinguish active fires were ongoing. The forest at Singelstorps fly (Figure 1; 56°59′25.0″ N 15°21′11.0″ E) was located to the eastern border of an area that had been burned in 2017. The new fire was lit at 13:00 on 27 June 2024. The observations ended at 20:00, totalling seven hours, when around half of the area had been burned. By the next day, the planned total of 27.8 ha had been burned, but an incipient thunderstorm prevented me from performing post‐fire observations.

## 3 Results

**FIGURE 2:**
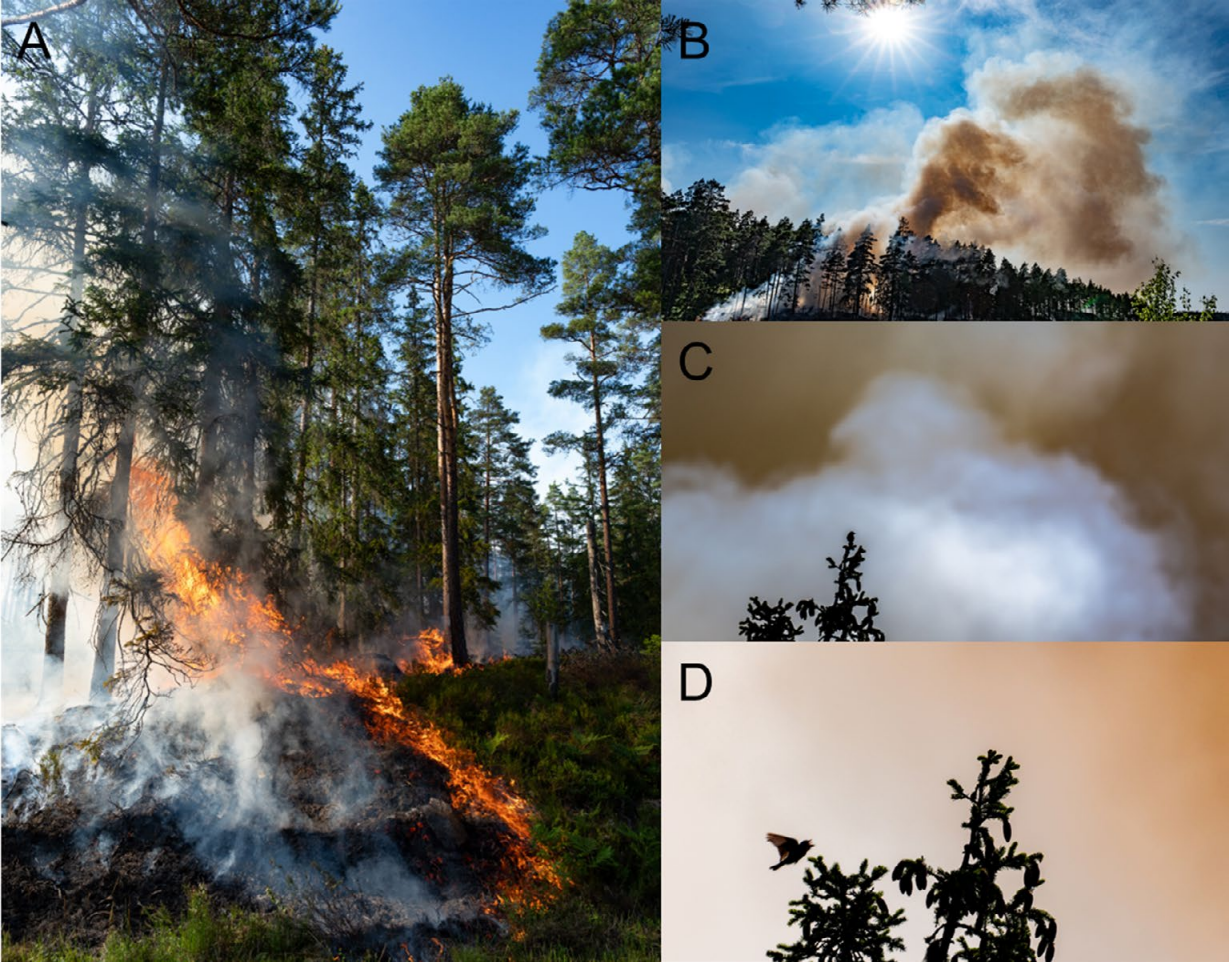
The prescribed fires at (A) Singelstorps fly and (B) Fjällmossen, where a Eurasian skylark frequently (C) perched in a tree top near the fire and (D) performed song flights through smoke.

The main results are presented in Table 1, showing the presence of seventeen species during or after the fires, and two species recorded only afterwards. To a large extent, this list of species can reflect a regular observation day at these locations. However, one would expect this list to be longer over a total of thirteen hours' observations on a typical day without prescribed burning. This suggests that detection probability was reduced due to smoke or that some species did avoid the fire, the dozens of people controlling it, or the drone flying over it (only at Singelstorps fly). A quantitative before‐after control‐impact (BACI) study would provide a more definite answer. Nonetheless, the behaviour of the present birds was compelling. While I did not observe them close to the fire, foraging near it, or foraging on burnt ground, they largely behaved normally. This is particularly illustrated by the singing behaviour of six species. Skylarks performed song flights through thick smoke (Figure 2), while robins, chaffinches, and tree pipits sang from treetops over or close to active fire. Blackbirds and blackcaps sang further from the fire and smoke. Of the species observed only the day after the fire, white‐tailed eagles seemed to merely pass by overhead, while black woodpeckers were active in the centre of the burnt area.

**TABLE 1:** Bird species observed or heard calling (O) and species observed and heard singing (S) at both locations during the prescribed burns and the day thereafter (Fjällmossen only).

Species	Fjällmossen		Singelstorps fly
	Fire	Next day	Fire
Black woodpecker ( *Dryocopus martius* )		O	
Blackbird ( *Turdus merula* )	S		S
Blackcap ( *Sylvia atricapilla* )			S
Blue tit ( *Cyanistes caeruleus* )	O	O	
Chaffinch ( *Fringilla coelebs* )	S	S	S
Common crossbill ( *Loxia curvirostra* )			O
Crested tit ( *Lophophanes cristatus* )			O
Cuckoo ( *Cuculus canorus* )	O	O	O
Eurasian jay ( *Garrulus glandarius* )			O
Eurasian nuthatch ( *Sitta europaea* )	O	O	
Eurasian siskin ( *Spinus spinus* )			O
Eurasian skylark ( *Alauda arvensis* )	S	S	
European robin ( *Erithacus rubecula* )	S		
Great spotted woodpecker ( *Dendrocopos major* )			O
Great tit ( *Parus major* )	O	O	O
Lesser whitethroat (*Curruca curruca*)	O		
Tree pipit ( *Anthus trivialis* )	S	S	
White‐tailed eagle ( *Haliaeetus albicilla* )		O	
Willow tit ( *Poecile montanus* )			O

## 4 Discussion

These descriptive observations suggests that many Swedish birds mostly ignore fire and smoke, while others may avoid it, although this remains to be established. Their response stands in stark contrast to the behaviour of birds from fire‐prone regions, where many species are attracted to the foraging opportunities offered by fire (as summarised in the Introduction). This is not a surprising result given the differences in fire regimes, but it is an important first step nonetheless to establish more robust comparisons of how fire ecology and its associated adaptations shape the immediate behavioural response of birds to active fire. There are also notable similarities, such that even in fire‐prone regions many bird species appear to ignore or avoid fire (see references in the Introduction), and some continue vocalising nearby (Dainezi and Ribeiro 2025). In the northeastern USA, the singing activity of birds is reduced proportionally to the density of wildfire smoke, mediated by species‐specific breeding phenology (Simamora et al. 2026). A similar pattern is yet untested but may hold for Swedish birds. The current study took place during the breeding season of the singing species (Billerman et al. 2026), which may have affected their responses to smoke (Simamora et al. 2026).

Future studies should systematically examine the abundance of birds before, during, and after prescribed fires in a controlled BACI design, and compare the results across gradients of fire regime characteristics. Raptors, insectivores, and generalists are all expected to exploit the foraging opportunities offered by fire, while granivores and scavengers may be more attracted to post‐fire landscapes. Naturally, this response is expected to be stronger in fire‐prone ecosystems, as suggested by the aforementioned studies in comparison to the current results. Birds still perish in fires despite their mobility, albeit at relatively low rates (Jolly et al. 2022; Tomas et al. 2021). It is therefore important to document whether repeated exposure to fire can affect the response of individual birds in their lifetime, which notably can also occur during migration. Learning to respond appropriately to fire may ameliorate negative effects of increasingly disrupted fire regimes. The plasticity of behaviour through cognition can buffer against adverse environmental change (Candolin et al. 2023; Greggor et al. 2016, 2020), and pyrocognition is likely no exception (Jacobs 2021; Jacobs et al. 2022). The senses used to detect fire are another underexplored aspect that likely fuels the ability to respond appropriately, or learn to do so (Candolin et al. 2023; Greggor et al. 2016, 2020; Michel et al. 2023; Nimmo et al. 2021). In that case, rescued animals could possibly be trained to avoid fire cues prior to their release, akin to anti‐predator training (Greggor et al. 2016, 2020). The surprising dearth of research on the immediate response of animals to active fire undercuts not only fundamental natural history and ethology, but also fire management and conservation.

## Author Contributions

**Ivo Jacobs:** conceptualization (lead), funding acquisition (lead), investigation (lead), methodology (lead), project administration (lead), resources (lead), visualization (lead), writing – original draft (lead), writing – review and editing (lead).

## Funding

This study was funded by the Vetenskapsrådet (Grant 2019‐03176).

## Conflicts of Interest

The author declares no conflicts of interest.

## Data Availability

All data is presented in the article.
